# Supplementary material for: Treatment with saturating dose of conventional anti-CD28 monoclonal antibody well tolerated in pig acute myocardial infarction model
Source: Front Immunol. 2026 Aug 11;17:1869344. doi: 10.3389/fimmu.2026.1869344 (PMC13503568; doi:10.3389/fimmu.2026.1869344)
Supplement: Supplementary file 1 [file Supplementaryfile1.pdf]

Treatment with saturating dose of conventional anti-CD28 monoclonal antibody well tolerated in pig acute myocardial infarction model

Anja Stadtmüller[1], Rebekka Grampp[1], Giulia Marianantoni[2], Florian Schnitter[1,3], Finja Becker[1], Könül Nuriyeva[2], Daniela Langenhorst[2], Leona Metka[2], Kira Günther[2], Lars Wiebusch[1], Kerstin H Mair[4], Nadine Gladow[3], Thomas Herrmann[2], Jan Rohr[5], Ulrich Hofmann[1,3], Anna Frey[1,3]*, Niklas Beyersdorf[2]*

[1] University Hospital Würzburg, Department of Internal Medicine I, Würzburg, Germany

[2] University of Würzburg, Institute for Virology and Immunobiology, Würzburg, Germany

[3] University Hospital Würzburg, Comprehensive Heart Failure Center, Würzburg, Germany

[4] University of Veterinary Medicine Vienna, Immunology Unit, Centre of Pathobiology, Department of Biological Sciences and Pathobiology, Vienna, Austria

[5] Albert-Ludwigs-University Freiburg, Institute of Immunodeficiency, Medical Center and Faculty of Medicine, Freiburg, Germany

* Equal contribution

ORCID IDs: Anja Stadtmüller (0009-0008-2854-2027), Florian Schnitter (0000-0002-7583-4224), Daniela Langenhorst (0009-0001-9144-6213), Kira Günther (0009-0004-5466-2509), Lars Wiebusch (0009-0006-3252-6551), Kerstin H. Mair (0000-0002-3538-8303), Nadine Gladow (0000-0003-0919-6348), Thomas Herrmann (0000-0002-1093-0194), Ulrich Hofmann (0000-0002-3681-2073), Anna Frey (0000-0002-2955-6753), Niklas Beyersdorf (0000-0002-9236-5415)

Supplementary Information

Supplemental Figure S1. Coronarangiography and macroscopically visible pathological changes in pig model of myocardial infarction. A) The LAD and its first lateral branch (blue star) were visualized by coronary angiography. The (upper) image shows the vessel before and (lower) during myocardial infarction induction with an inflated occlusion balloon. **B**) The lighter infarcted area of the pig hearts can be identified macroscopically (colored line) – one example of an explanted heart of a control mAb and of a mAb 3D11-treated animal. **C**) A slice from the left ventricle to illustrate the sampled areas. The three zones (infarct core, border zone and remote myocardium) can be differentiated macroscopically. **D**) For each heart slice the infarcted area was first determined as % area of the whole slice which was converted into weight by multiplying % area with the weight of the slice. The sum of the weights of the infarcted areas of all slices was then divided by the total heart weight to calculate the proportion of infarcted heart muscle tissue. Unpaired two-sided Mann-Whitney test. ** $p < 0.01$. **E**) Body temperature (rectal) was measured on day 0 before MI induction and on day 3, i.e. after antibody had been infused over 30 min. Grey arrows: This pig died immediately after completion of the infusion with signs of cardiogenic shock.

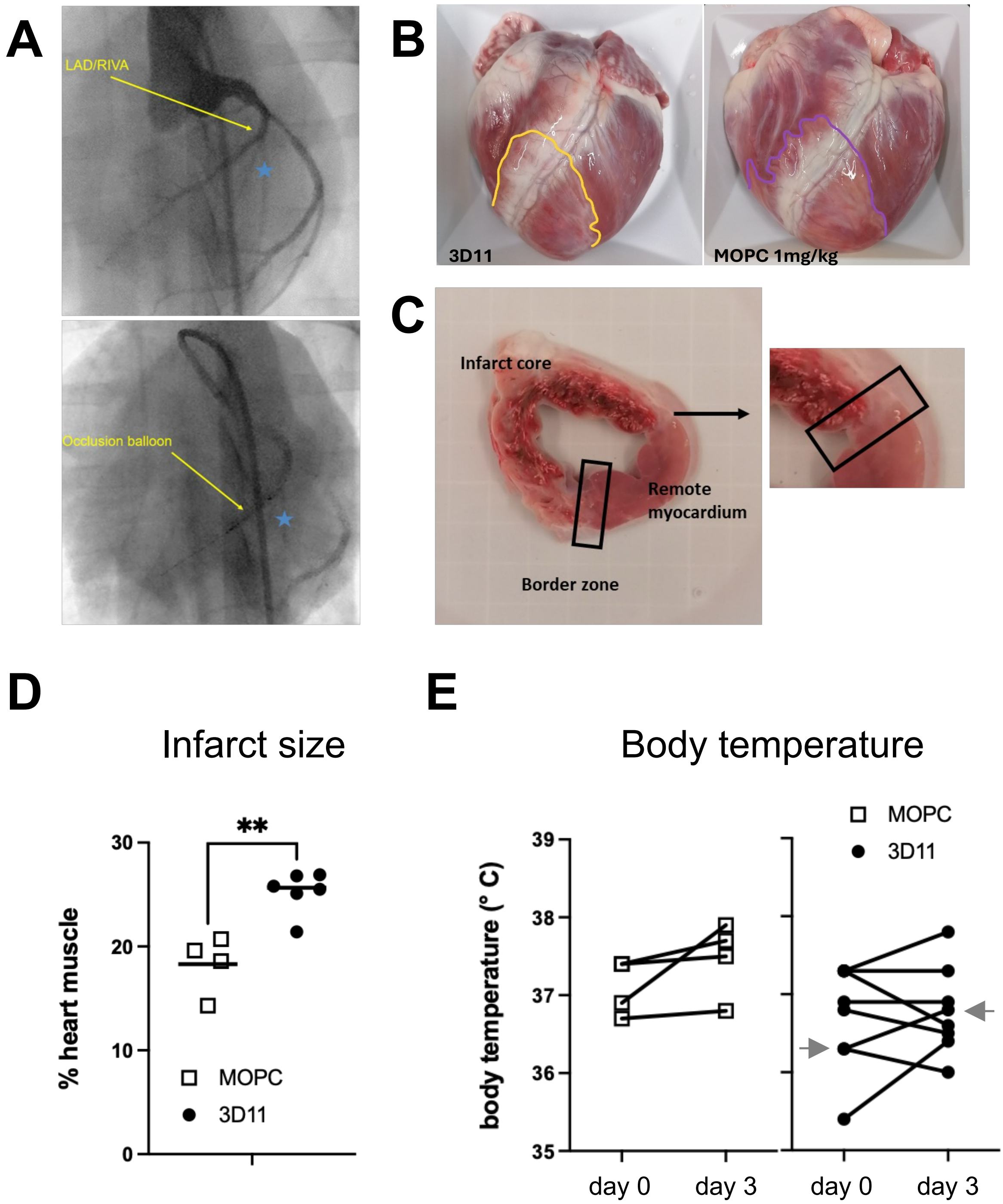

A
LAD/RIVA
Occlusion balloon
B
3D11
MOPC 1mg/kg
C
Infarct core
Remote myocardium
Border zone
D
Infarct size
% heart muscle
**
MOPC
3D11
E
Body temperature
body temperature (° C)
MOPC
3D11
day 0
day 3
day 0
day 3

Supplemental Figure S2.

Gating strategy to monitor antibody infusion and T cell subsets in peripheral blood. A) Identification of $CD4^+$ and $CD8^+$ T cells in peripheral blood. **B**) detection of infused mAb 3D11 on $CD4^+$ T cells on day 7 (black) compared to day 0 (grey). **C**) CD28 expression on peripheral blood $CD4^+$ T cells before (grey) and seven days after MI induction (black). Dashed lines show controls without primary and secondary antibody for detection of CD28 expression. **D**) Detection of TruCount beads (left) and leukocyte gating (right). **E**) Among leukocytes, $CD45^+$ $CD3^+$ T cells were further gated according to CD4 and CD8 expression to determine absolute numbers of $CD3^+$ $CD45^+$ $CD4^+$ and $CD8^+$ T cells per μl of blood. **F**) Detection of $CD25^+$ $Foxp3^+$ Treg among gated $CD4^+$ T cells. **G**) Ki-67 versus Foxp3 expression by $CD4^+$ T cells of peripheral blood.

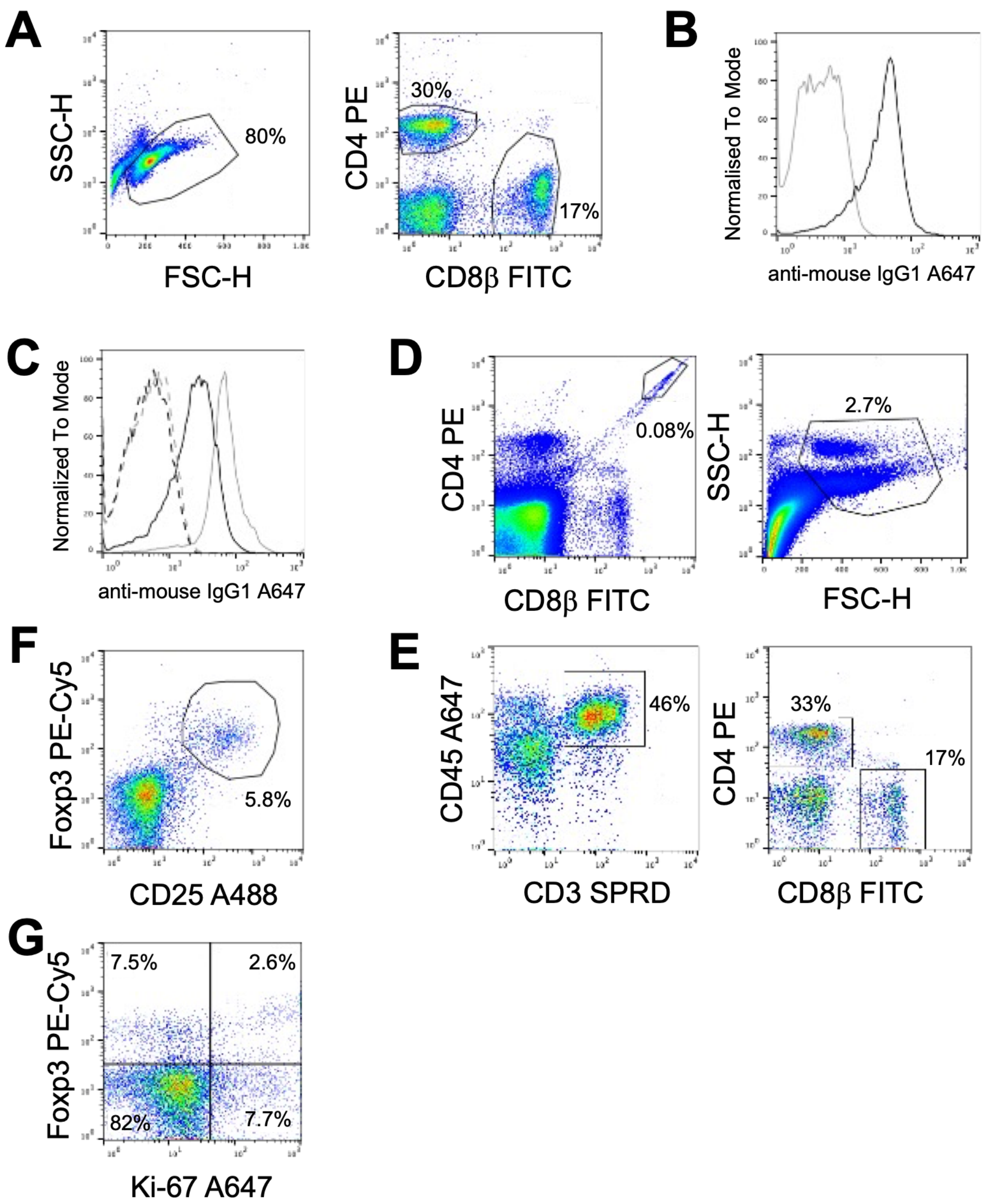

Supplemental Figure S3.

Gating strategy for detecting heart-infiltrating Treg and Tconv. A) Identification of lymphocytes. **B**) Gating of CD45$^+$ CD4$^+$ T cells. Anti-CD45 FITC was used together with anti-FITC magnetic beads (Miltenyi) to isolate leukocytes from digested heart tissue. **C**) Detection of CD25$^+$ Foxp3$^+$ Treg among gated CD4$^+$ T cells. In addition to anti-CD45 FITC used for isolation of leukocytes, the cells were stained with anti-CD25 FITC. **D**) Ki-67 versus Foxp3 expression by heart-infiltrating CD4$^+$ T cells.

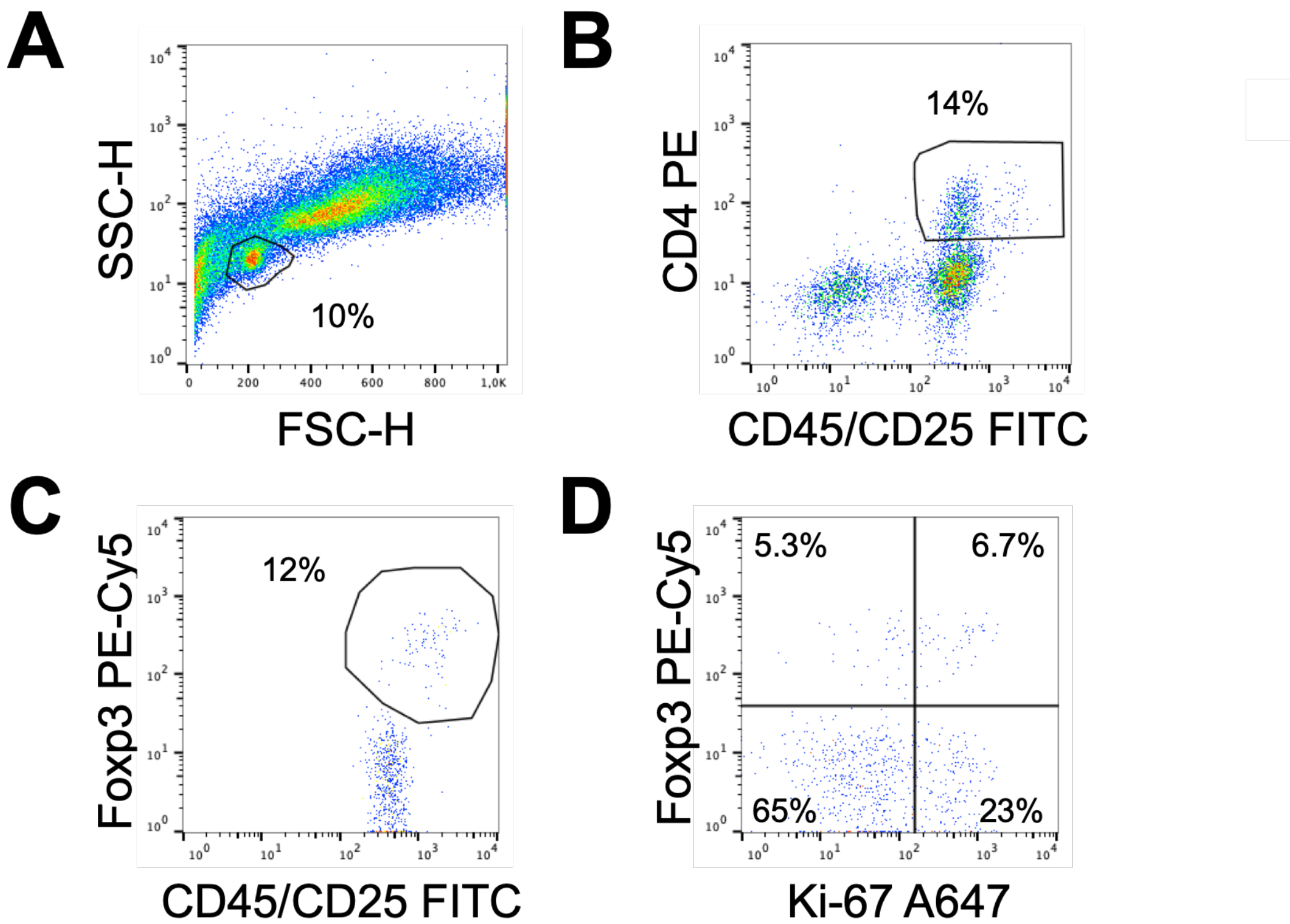

Supplemental Figure S4.

Schematic overview of immunofluorescence assays for detecting infused mAb 3D11 bound to and overall expression of CD28 by CD4$^+$ T cells. **A**) Bound mAb 3D11 was detected with a secondary anti-mouse IgG antibody. **B**) To determine CD28 expression blood, spleen or mediastinal lymph node cells were first incubated *ex vivo* with mAb 3D11 in saturation. After multiple washes and transfer of cells to another well, bound mAb 3D11 was again visualised using anti-mouse IgG.

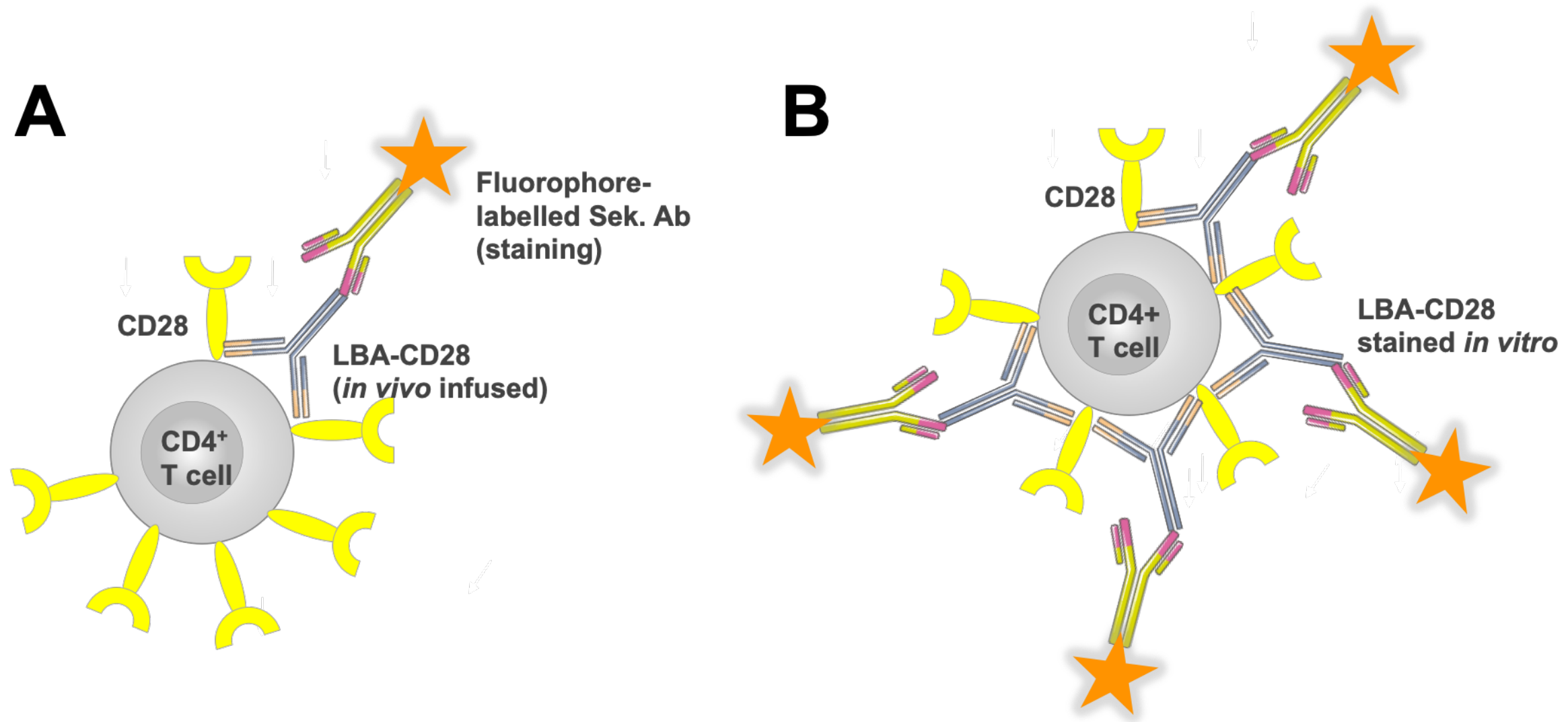

Supplemental Figure S5.

Longitudinal analysis of T cell subsets in peripheral blood after infusion of mAb 3D11 *in vivo.* mAb 3D11 was infused on day 3 after MI induction. **A**) Absolute number of total T cells, **B**) $CD4^+$ T cells and **C**) $CD8^+$ T cells per μl of blood on the day of MI induction as well as on days 3 and 7 post MI induction. **D**) Proportion of $CD4^+$ T cells among peripheral-blood leukocytes. **E**) Proportion of $CD25^+$ $Foxp3^+$ Treg among $CD4^+$ T cells. **F**) Proportion of Ki-67^+ cells among Treg and **G**) Tconv. Repeated measures two-way ANOVA followed by Tukey's post-hoc test. $0.05 \leq p < 0.1$ are shown as numbers. * $p < 0.05$, ** $p < 0.01$, *** $p < 0.001$.

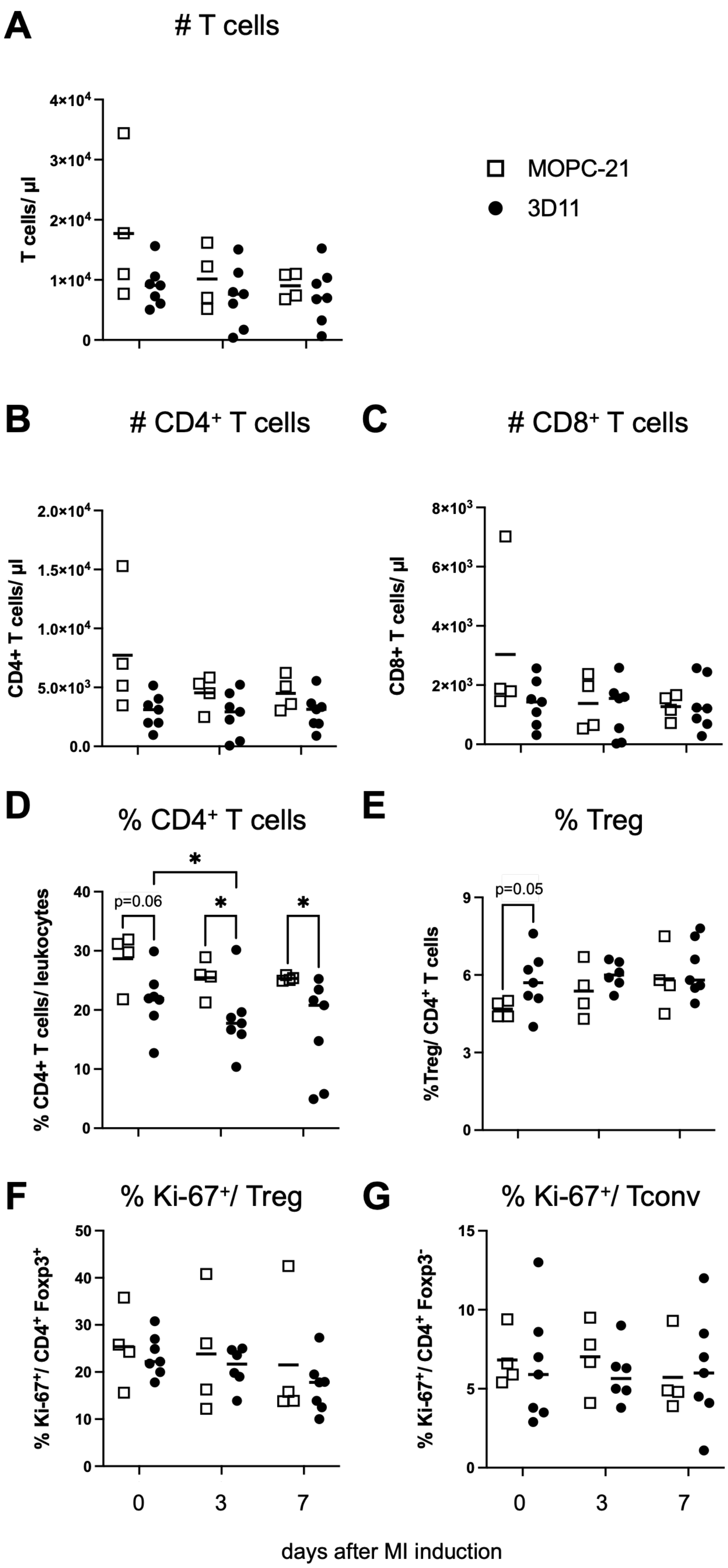

A
T cells
T cells/ µl
4×10^4
3×10^4
2×10^4
1×10^4
0
MOPC-21
3D11
B
CD4+ T cells
CD4+ T cells/ µl
2.0×10^4
1.5×10^4
1.0×10^4
5.0×10^3
0.0
C
CD8+ T cells
CD8+ T cells/ µl
8×10^3
6×10^3
4×10^3
2×10^3
0
D
% CD4+ T cells
% CD4+ T cells/ leukocytes
p=0.06
*
40
30
20
10
0
E
% Treg
%Treg/ CD4+ T cells
p=0.05
9
6
3
0
F
% Ki-67+/ Treg
% Ki-67+/ CD4+ Foxp3+
50
40
30
20
10
0
G
% Ki-67+/ Tconv
% Ki-67+/ CD4+ Foxp3-
15
10
5
0
0
3
7
days after MI induction

Supplemental Figure S6.

mAb 3D11 infusion did not have an obvious impact on the CD4⁺ T cell compartment in spleen and mediastinal lymph nodes. mAb 3D11 was infused on day 3 after MI induction. Spleen and mediastinal lymph nodes (med. LN) were analysed on day 7. **A**) Proportion of $CD4^+$ T cells among leukocytes. **B**) Proportion of $CD25^+$ $Foxp3^+$ Treg among $CD4^+$ T cells. **C**) Proportion of Ki-67^+ cells among Treg and **D**) Tconv. Repeated measures two-way ANOVA followed by Tukey's post-hoc test. * $p < 0.05$, *** $p < 0.001$, **** $p < 0.0001$.

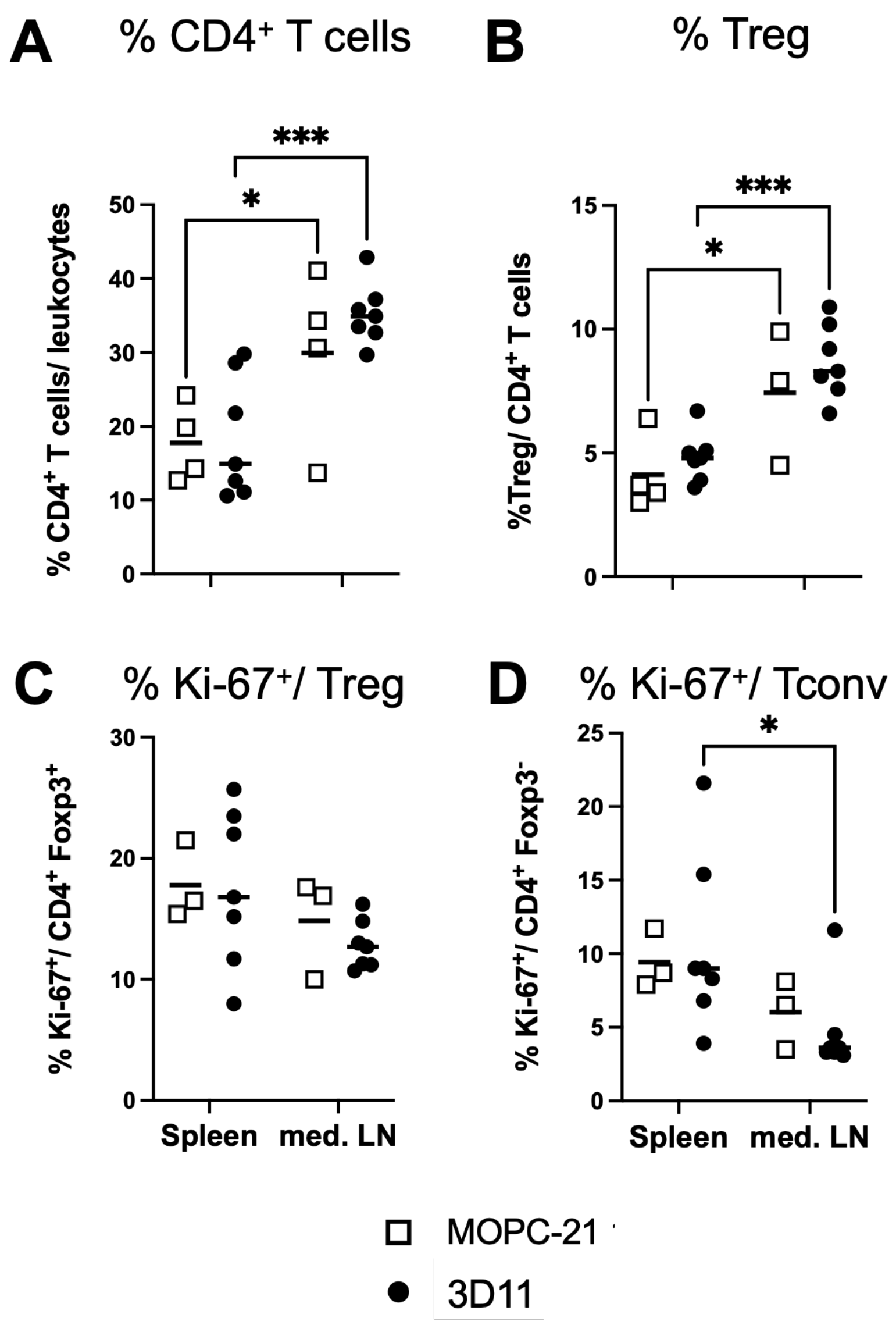

Supplemental Table 1

List of monoclonal primary antibodies used for flow cytometry (FC) analysis of whole blood samples

Antigen	Conjugate	Isotype	Clone	Species	Format	Source	Concentration
CD3ε	SPRD	IgG1	PPT3	mouse:pig	labeled	southern biotech	1:500
CD4α	PE	IgG2b	74-12-4	mouse:pig	labeled	BD	1:100
CD8β	FITC	IgG1	PPT23	mouse:pig	labeled	Biorad	1:400
CD45	A647	IgG1	K252.1E4	mouse:pig	labeled	Biorad	1:15

Supplemental Table 2

List of monoclonal primary antibodies used for flow cytometry (FC) analysis of PBMC, spleen, lymph node, and heart samples

**Antigen**	**Conjugate**	**Isotype**	**Clone**	**Reactivity**	**Format**	**Source**	**Concentration**
none	/	all	polyclonal	mouse:unknown	unlabelled	Sigma Aldrich	1:50
pig CD4α	PE	IgG2b	74-12-4	mouse:pig	labelled	BD	1:100
pig CD8β	FITC	IgG1	PPT23	mouse:pig	labelled	Biorad	1:400
Foxp3	PE-Cy5	IgG2a	FJK-16s	rat:mouse	labelled	Thermo Fisher	1:100
Ki67	A647	IgG1	B56 (RUO)	mouse:human	labelled	BD	1:300
pig CD25	A488	IgG1	K231.3B2	mouse:pig	labelled	Biorad	1:200
mouse IgG	A647	IgG1	RM61-1	rat:mouse	labelled	BioLegend	1:2000
CD28	/	IgG_1	3D11	mouse:pig	unlabelled	in vivo Biotech	1:200
CD28	/	IgG_1	4D12	mouse:pig	unlabelled	in vivo Biotech	1:100

Supplemental Table 3

Flow cytometry staining workflow

Row / Panel	20 min on ice	Wash 1	15 min on ice	Wash 2	15 min on ice	15 min on ice	Wash 3	30 min on ice	Wash 4	45 min at RT (in the dark)	Wash 5
**1**	---	1x, change well; 1x	amIg-A647	1x	normal mouse Ig (nmIgG)	CD8β-FITC CD4-PE	2x				
**2**	4D12	1x, change well; 1x	amIg-A647	1x	normal mouse Ig (nmIgG)	CD8β-FITC CD4-PE	2x				
**3**	3D11	1x, change well; 1x	amIg-A647	1x	normal mouse Ig (nmIgG)	CD8β-FITC CD4-PE	2x				
**4**	---		---	1x	normal mouse Ig (nmIgG)	CD8β-FITC CD4-PE CD27-APC	2x				
**5**				1x	normal mouse Ig (nmIgG)	CD25-A488 CD4-PE	1x PBS	Fix/Perm	1x	FoxP3-PE-Cy5 Ki67-A647	2x
**6**				1x	normal mouse Ig (nmIgG)	CD4-PE	1x PBS	Fix/Perm	1x	FoxP3-PE-Cy5 Ki67-A647	2x
**7**				1x	normal mouse Ig (nmIgG)	CD4-PE	1x PBS	Fix/Perm	1x	---	2x
**Volumes**	50 µl	150 µl FACS-buffer	50 µl	150 µl FACS-buffer	25 µl	25 µl	150 µl	100 µl	100 µl Perm-buffer	50 µl	150 µl FACS-buffer

Supplemental Videos

All videos are of one pig receiving 1 mg/ kg body weight mAb 3D11 on day 3 post MI.

Video 1

day 0 - parasternal long axis

Video 2

day 0 - parasternal short axis

Video 3

day 0 - subxyphoidal-apical

Video 4

day 7 - parasternal long axis

Video 5

day 7 - parasternal short axis

Video 6

day 7 - subxyphoidal-apical
